# A Reproducible Python-Based Computational Pipeline for Real-Time Ingestion, Advanced Analysis, and Dynamic Reporting of Public Health Data: A Systems Validation Study

**DOI:** 10.7759/cureus.103008

**Published:** 2026-02-05

**Authors:** Pavan Kumar Krishnapur, Chaitra CM, Arjun B, Ananthakrishnan M

**Affiliations:** 1 Community and Family Medicine, All India Institute of Medical Sciences, Bhopal, Bhopal, IND

**Keywords:** bioinformatics, data managment, public health informatics, python programming, workflow automation

## Abstract

Background

The analysis of large-scale public health data is crucial for evidence-based policymaking, but conventional workflows involving manual data handling and static reporting are inefficient and lack reproducibility. There is a need for automated tools that bridge the gap from live data sources to sophisticated, dynamically generated insights.

Objective

To design, implement, and validate a fully automated Python (Python Software Foundation, Wilmington, DE, USA) pipeline for real-time application programming interface (API)-based ingestion of existing datasets, analysis, and dynamic report generation in public health informatics.

Methods

We developed a lightweight, Python-only, Word-report-oriented pipeline using packages including Pandas, scikit-learn, statsmodels, and python-docx. The pipeline ingests data from public APIs with automated retries, performs preprocessing, calculates composite health scores, applies K-means clustering (k = 3) for state stratification, and performs correlation analysis. A custom rule-based engine generates dynamic textual interpretations based on statistical results. The final output is a programmatically constructed Microsoft Word (Microsoft Corporation, Redmond, WA, USA) document containing narrative, tables, and embedded figures. The pipeline was tested using India's Health and Family Welfare Statistics 2015 dataset via the data.gov.in API.

Results

The pipeline executed successfully in approximately 95 seconds, ingesting 37 records. It generated a composite health score, identifying Meghalaya as the top performer (score: 100.0). K-means clustering stratified states into three distinct performance tiers. Correlation analysis revealed a significant negative association between sub-health centre (SHC) infrastructure and specialist availability (r = -0.446, p = 0.01), as well as between 24×7 service availability and auxiliary nurse midwife (ANM) staffing (r = -0.358, p = 0.05), highlighting a systemic disconnect between capital investment in facilities and human resource allocation. A complete Word report including these findings, figures, and tables was automatically generated.

Conclusion

This automated framework provides a robust, efficient, and reproducible solution for transforming raw public health data into actionable insights and can significantly accelerate data-driven discovery and reporting in public health and bioinformatics. This study validates a computational framework for automated public health data analysis and reporting.

## Introduction

Public health datasets, including national health surveys, disease surveillance systems, and electronic health records (EHRs), play a central role in understanding population health trends, monitoring health system performance, and informing evidence-based policy decisions. With the expansion of digital health infrastructure and open data initiatives, the volume, variety, and update frequency of such datasets have increased substantially, creating opportunities for near real-time monitoring and advanced analytics.

Despite this growth in data availability, the transformation of raw public health data into actionable insights remains operationally challenging. Public health analysts frequently work under time constraints that require repeated analyses as new data become available. Conventional analytical workflows typically involve manual steps, such as downloading datasets, cleaning and restructuring data, executing analyses, generating visualisations, and compiling narrative reports. These processes are labour-intensive, error-prone, and highly dependent on individual analysts, making them difficult to scale, reproduce, or standardise across datasets and time points [1].

Even when computational tools such as Jupyter Notebooks (RStudio, PBC, Boston, MA, USA) or R Markdown are employed, human execution remains necessary at multiple stages of the workflow. Fragmentation across scripts, software environments, and manual decision points limits the ability to perform consistent, repeatable analyses, particularly in routine surveillance or continuous monitoring settings. As a result, analytical outputs may vary between runs, updates are delayed, and reproducibility is compromised.

Several computational approaches and analytical tools have been developed to support components of public health data analysis, including data pre-processing, statistical modelling, and visualisation. However, most existing solutions address isolated stages of the analytical pipeline and require substantial manual coordination to integrate data ingestion, pre-processing, analysis, interpretation, and reporting. Fully automated, end-to-end pipelines capable of executing the entire analytical cycle with minimal human intervention remain limited within the public health informatics literature [2].

To address these challenges, this study presents a reproducible, fully automated Python-based pipeline that integrates real-time API-based ingestion of existing datasets, structured pre-processing, advanced analytical methods, dynamic narrative interpretation, and automated report generation within a single workflow [3]. The framework is designed to operate through a single-command execution, minimising manual intervention while ensuring reproducibility and scalability. The pipeline is dataset-agnostic and intended for reuse across diverse public health contexts, supporting routine monitoring, exploratory analysis, and standardised reporting.

The functionality of the pipeline is demonstrated using a publicly available national health dataset obtained from the Government of India’s open data portal. This dataset is used solely to validate and illustrate the pipeline's operational capabilities rather than to draw substantive epidemiological conclusions. By automating the complete analytical workflow, this framework aims to reduce manual workload, improve reproducibility, and enable timely, data-driven decision-making in public health practice.

## Materials and methods

Study design

This study is a computational methods and systems validation study, demonstrating the design, implementation, and validation of an automated pipeline for public health data analysis. The work focuses on system architecture, workflow automation, and reproducibility rather than hypothesis-driven epidemiological inference. The pipeline was developed to demonstrate a fully automated, end-to-end analytical workflow capable of transforming raw public health data into structured analytical outputs and reports with minimal human intervention.

Architecture overview

The pipeline follows a five-stage architecture comprising data ingestion, data pre-processing, data analysis, data interpretation, and data reporting. This abstraction was intentionally designed to be dataset-agnostic, enabling different analytical techniques to be incorporated into the analysis stage depending on the use case. The pipeline is implemented as a modular, object-oriented Python framework and executes the complete workflow through a single command, ensuring reproducibility and consistency across runs.

**Figure 1:**
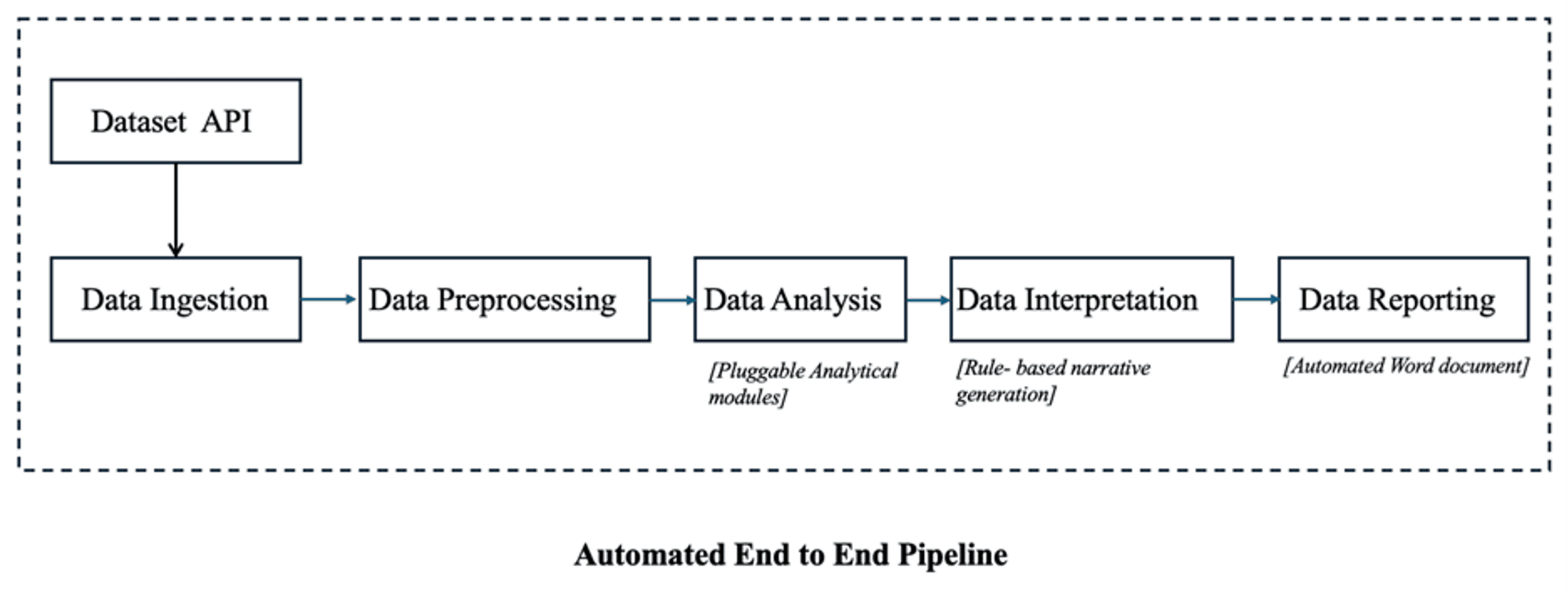
Conceptual architecture of the automated end-to-end data analysis pipeline. API: application programming interface.

Each stage of the pipeline performs a clearly defined function while remaining fully integrated within the overall workflow. Specific analytical methods applied during the analysis stage represent example implementations rather than fixed components of the pipeline. This design allows the framework to be reused and extended across diverse public health datasets and analytical contexts. The overall architecture and flow of the automated pipeline are illustrated in Figure 1.

The pipeline follows a five-stage, dataset-agnostic workflow comprising data ingestion, data pre-processing, data analysis, data interpretation, and data reporting. Analytical methods are implemented as pluggable modules within the analysis stage, while interpretation and reporting are performed automatically through rule-based narrative generation and programmatic document creation.

Data source and demonstration dataset

The pipeline was demonstrated using the Health and Family Welfare Statistics 2015 dataset accessed via the Government of India's open data application programming interface (API) available at data.gov.in. The dataset consists of aggregated, state-level public health indicators derived from nationally representative sources.

The dataset was used solely to demonstrate and validate the operational capabilities of the pipeline. The analytical outputs generated are illustrative in nature and were not intended to produce definitive epidemiological conclusions.

Data ingestion

The data ingestion stage is responsible for automated retrieval of structured data from public APIs. Data were fetched programmatically in comma-separated values (CSV) format using API requests with built-in pagination handling to ensure complete retrieval of all available records. To improve robustness, an automated retry mechanism with exponential backoff was implemented to handle transient network failures or unstable servers.

All retrieved data were stored in structured data frames, providing a standardised input for downstream processing and enabling traceability of the ingestion process.

Data pre-processing

The pre-processing stage performs systematic cleaning and standardisation of raw input data. This includes harmonising column names, handling missing values, converting variables to appropriate numeric formats, and removing aggregate or summary rows to ensure analytical consistency. Missing values were imputed using the median to reduce sensitivity to outliers. Continuous variables were converted to numeric types and retained without transformation prior to normalisation in downstream analyses. Feature engineering steps were applied programmatically to derive additional variables required for analysis, such as regional groupings and calculated indicators. 

Data analysis

The data analysis stage serves as a flexible analytical layer within the pipeline. Analytical methods are implemented as modular components that can be enabled, modified, or replaced depending on the dataset and analytical objectives.

For the purpose of demonstrating the pipeline, the following analysis was done on the example dataset. Composite scoring, all selected indicators were standardised using z-score normalisation (StandardScaler) to ensure comparability across heterogeneous scales, and the composite score was calculated as an unweighted mean of normalised indicators before linear rescaling to a 0-100 range. Unsupervised clustering, the number of clusters (k = 3) was chosen a priori for interpretability rather than post hoc optimisation, Euclidean distance was used as the similarity metric, and a fixed random seed ensured deterministic cluster assignments. Correlation analysis and Pearson correlation analysis assumed linear relationships and approximate normality of variables at the state-aggregated level. Statistical testing, all t-tests were exploratory in nature, employed Welch correction where applicable, and no adjustment for multiple comparisons was performed.

These analyses are specific for this particular dataset and have been chosen for illustrating the capabilities of the pipeline rather than to draw epidemiological inferences from it. The analysis module of the pipeline can be adapted and modified based on the dataset at hand and analytical requirements.

Data interpretation

The data interpretation stage converts analytical outputs into structured, context-aware narrative summaries. A rule-based interpretation engine was implemented using conditional logic to evaluate analytical results, including statistical significance, rankings, and clustering assignments.

Based on predefined rules, the pipeline automatically generates textual interpretations describing key findings, including identification of top- and bottom-performing units and interpretation of statistical results. This stage reduces reliance on manual report writing and ensures consistent interpretation across repeated analyses.

Data reporting

The final stage of the pipeline programmatically generates a formatted Microsoft Word (Microsoft Corporation, Redmond, WA, USA) report containing narrative text, tables, and figures. Document structure, headings, and content are created automatically based on pipeline outputs. Analytical tables are derived directly from processed data frames, and visualisations generated during analysis are embedded within the report. Each report is saved with a unique timestamped filename to prevent overwriting and to support version tracking. The entire report is produced without manual editing following pipeline execution.

Software environment and reproducibility

All analyses were performed using Python version 3.10 (Python Software Foundation, Wilmington, DE, USA) within a standardised scientific computing environment. The pipeline relies on widely used open-source libraries for data manipulation, statistical analysis, visualisation, and document generation. The modular design and single-command execution ensure deterministic and reproducible outputs when run on identical inputs. The core software libraries used across different stages of the pipeline and their functional roles are summarised in Table 1.

**Table 1:** Core software libraries used in the pipeline and their functional roles.

Pipeline stage	Software library/tool	Functional role	References
Data ingestion	requests, pandas	Programmatic API access, pagination handling, and structured data retrieval	[3,4]
Data preprocessing	pandas, numpy, re	Data cleaning, standardization, handling missing data, and feature engineering	[4-6]
Data analysis	scikit-learn, scipy	Normalisation, composite scoring, clustering, correlation analysis, and statistical testing	[7,8]
Data visualisation	Matplotlib, seaborn	Generation of plots and graphical summaries	[9,10]
Data interpretation	Python conditional logic	Rule-based generation of context-aware narrative interpretations	[5]
Data reporting	python-docx	Automated creation of formatted Microsoft Word reports	[11]
Reproducibility	Python 3 environment	Deterministic execution and reproducible workflow	[5]

Validation strategy

Validation focused on numerical consistency and logical correctness of the pipeline. Pipeline validation was performed using a predefined set of reproducibility and consistency checks. These included verification of row counts before and after pre-processing, confirmation of numeric type consistency following data transformation, replication of composite score calculations using independent spreadsheet-based methods, and cross-validation of statistical test outputs using external statistical software (Jamovi v2.6.44, The jamovi project (2025), jamovi (Version 2.6) Computer Software).

## Results

Pipeline execution and performance

The automated pipeline executed successfully from data ingestion to report generation using the demonstration dataset obtained via the data.gov.in API. All five stages of the workflow-data ingestion, pre-processing, analysis, interpretation, and reporting-were completed without manual intervention following single-command execution. The complete pipeline run was completed in approximately 95 seconds. After the removal of aggregate summary rows, 37 state and union territory records were included in the analysis.

Data ingestion and pre-processing outputs

Structured CSV data were retrieved across multiple API calls and consolidated into a unified dataset. Pre-processing steps, including column standardisation, handling of missing values, numeric type conversion, and feature derivation, were executed deterministically and produced a cleaned dataset suitable for downstream analysis. Repeated executions using identical inputs yielded identical pre-processed outputs.

Example analytical outputs

As an illustration of the pipeline’s analytical capabilities, multiple analytical modules were applied to the demonstration dataset during the data analysis stage.

**Figure 2:**
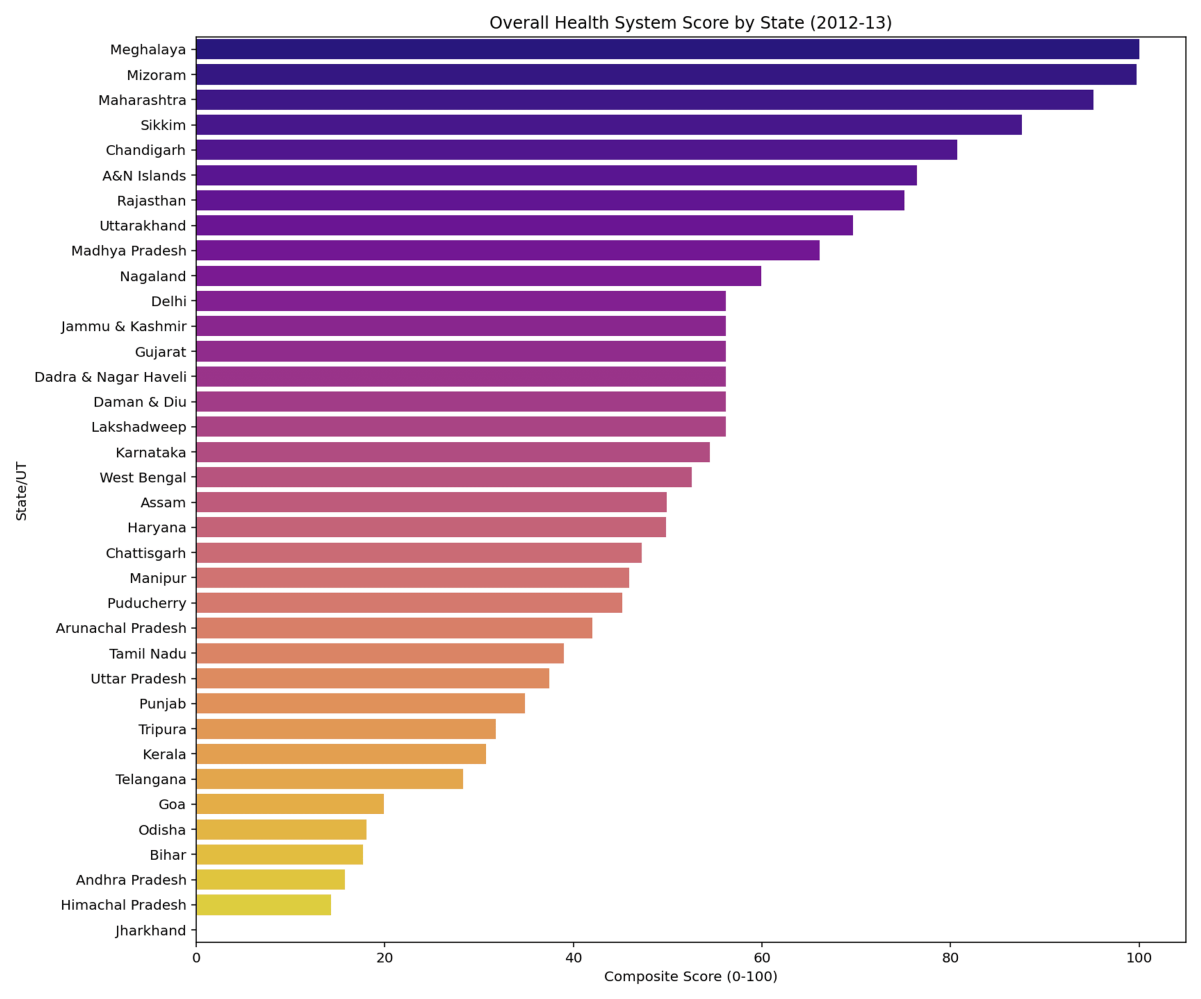
State-wise ranking based on the composite health system score (2012–2013).

A composite health system score was generated by z-score normalisation and aggregation of selected indicators, followed by linear rescaling to a 0-100 range. State-wise rankings based on this composite score were produced automatically, with Meghalaya achieving the highest composite score (100.0). Figure 2 presents the state-wise ranking based on the composite health system score generated by the pipeline.

The figure illustrates relative state performance derived from normalised and aggregated health system indicators. Scores were generated automatically as part of the pipeline’s analytical workflow.

**Figure 3:**
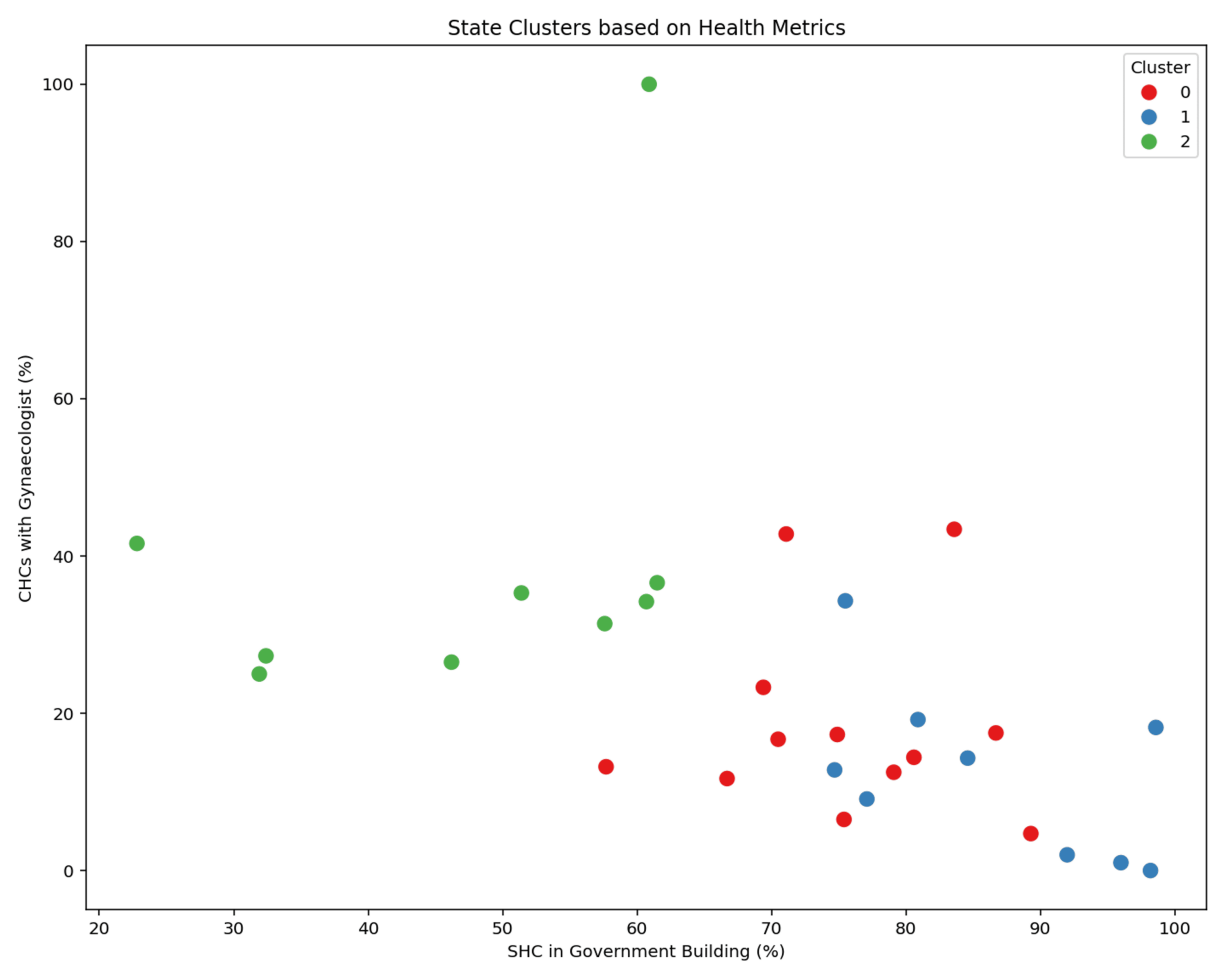
State clustering based on composite health system indicators. SHC: sub-health centre.

Unsupervised clustering using the K-means algorithm (k = 3) stratified states into three distinct performance groups based on composite indicator profiles, as illustrated in Figure 3.

States were grouped into three clusters using K-means clustering to demonstrate the pipeline’s capability for unsupervised exploratory analysis.

**Figure 4:**
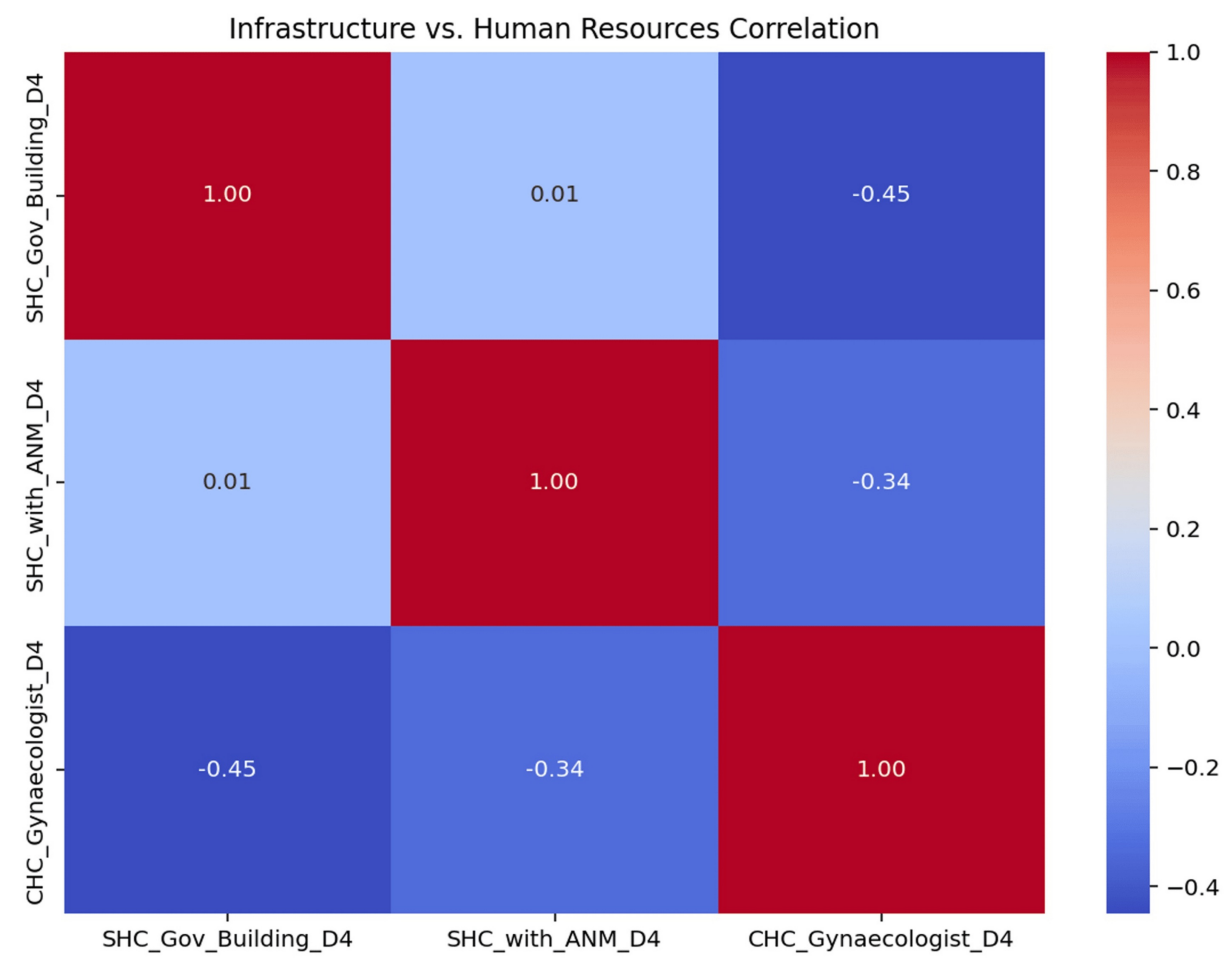
Correlation between selected infrastructure and human resource indicators. SHC: sub-health centre, ANM: auxiliary nurse midwife.

Correlation analysis revealed a significant negative association between SHC infrastructure and specialist availability (r = -0.45, p = 0.01), as well as between 24x7 service availability and ANM staffing (r = -0.34, p = 0.05). Conversely, SHC infrastructure showed no significant correlation with ANM presence (r = 0.01, p = 0.90), highlighting a gap between capital investment in facilities and human resource allocation. The correlation matrix is illustrated in Figure 4.

Paired t-testing comparing primary health centre (PHC) 24×7 service availability between District Level Household and Facility Survey (DLHS-3) and DLHS-4 at the national level showed no statistically significant change (p = 0.23).

These analytical results are presented as example outputs to demonstrate the flexibility of the pipeline and do not represent definitive epidemiological inferences.

Automated interpretation outputs

Analytical results were automatically translated into narrative summaries by the dynamic interpretation engine. The system programmatically evaluated rankings, clustering assignments, and statistical test outcomes using predefined conditional logic and generated context-aware textual interpretations.

The interpretation stage consistently identified key patterns, such as relative performance rankings and the presence or absence of statistically significant differences, without manual input. This process ensured standardised and reproducible interpretation of results across pipeline runs (see Appendix).

Automated report generation

Following analysis and interpretation, the pipeline automatically generated a structured Microsoft Word document containing narrative summaries, analytical tables, and embedded visualisations. The report was created programmatically using predefined document templates and populated dynamically with pipeline outputs.

The generated document included section headings, formatted tables derived from processed data frames, and figures generated during analysis. Each report was saved with a unique timestamped filename to support version tracking and prevent overwriting.

Output consistency and validation

Pipeline outputs were validated through manual cross-checking against independently generated results using spreadsheet software and statistical tools. Numerical outputs, rankings, and statistical test results were found to be consistent with independently computed values. Repeated executions of the pipeline on identical inputs produced consistent analytical results and identical report structures, confirming deterministic behaviour and reproducibility of the workflow.

## Discussion

This study presents and validates a modular, automated Python-based pipeline designed for end-to-end ingestion, pre-processing, analysis, interpretation, and reporting of public health datasets. Importantly, this work is positioned as a computational systems and methods validation study rather than an epidemiological investigation. The primary contribution lies in demonstrating the technical feasibility, internal consistency, reproducibility, and practical utility of a single-command analytics pipeline capable of transforming raw, publicly available health data into structured analytical outputs and narrative reports with minimal manual intervention. 

The need for reproducible, scalable, and automated analytical workflows in public health and healthcare analytics is well recognised. Prior work has highlighted how fragmented data pre-processing, ad hoc statistical scripting, and undocumented analytical decisions undermine transparency and reproducibility in health research [1,12]. While several frameworks and libraries address individual components of the analytical lifecycle--such as data manipulation (pandas), statistical modelling (SciPy), and machine learning (scikit-learn)--integration across the entire workflow often remains user-dependent and inconsistently implemented. Recent large-scale frameworks such as ehrapy have demonstrated the power of modular, end-to-end pipelines for exploratory analysis of complex electronic health record data, including pre-processing, normalisation, clustering, and downstream statistical inference [13]. However, such platforms are primarily designed for multi-modal EHR data and advanced phenotyping tasks. In contrast, the pipeline presented in this study focuses specifically on public health tabular datasets accessed via government APIs, emphasising rapid deployment, minimal configuration, and automated narrative reporting, which are particularly relevant in operational public health and training contexts.

Using the Health & Family Welfare Statistics 2015 dataset solely as a demonstration input, the pipeline successfully executed all intended modules, including data ingestion with automated retry logic, structured pre-processing, feature engineering, clustering, correlation analysis, and report generation. The pre-processing strategies-standardised column normalisation, numeric coercion, exclusion of aggregate records, and derived indicator generation-reflect best practices widely recommended for secondary analysis of administrative health data [2,14]. Clustering using K-means (k = 3) produced internally coherent state-level groupings based on composite health indicators. While clustering has been widely used for exploratory stratification in health analytics, this study does not interpret clusters substantively; instead, clustering is employed as a functional validation step to confirm correct execution, stability, and interpretability of the pipeline’s analytical modules. Similarly, Pearson correlation coefficients were computed to validate statistical operations, with representative outputs demonstrating expected directional relationships between indicators. These correlations are presented as computational outputs, not as causal or epidemiological findings.

Reproducibility remains a persistent challenge in computational health research, particularly when analytical pipelines are manually assembled or poorly documented. By encapsulating the entire workflow within a class-based, object-oriented architecture and executing it via a single command, the proposed pipeline addresses key concerns raised in reproducible research literature [1]. Explicit software dependencies, deterministic pre-processing steps, and automated report generation reduce analyst-induced variability and enhance auditability. This pipeline is particularly suited for training, rapid situational analysis, and standardised reporting within public health institutions. Government analysts, academic trainees, and program evaluators frequently encounter repetitive analytical tasks that differ mainly in the input datasets rather than in the analytical logic. Automating these workflows can substantially reduce turnaround time, minimise errors, and improve consistency across reports. Prior studies have emphasised that such automation can enhance decision-support capacity without replacing domain expertise [15].

Limitations

The pipeline was demonstrated using a single publicly available, aggregated dataset, and its performance was not benchmarked across multiple datasets or real-time surveillance environments. The rule-based interpretation engine relies on predefined logic, which may require customisation or extension for different analytical contexts or domain-specific reporting needs. While the pipeline supports automated statistical testing, it does not replace expert judgment and should be viewed as a decision-support tool rather than an autonomous decision-making system.

Future directions

Future work may focus on extending the pipeline to support additional data sources, including electronic health records and real-time surveillance feeds. Enhancements to the interpretation engine using adaptive or machine learning-based approaches may further improve flexibility and contextual awareness. Integration with additional output formats, such as dashboards or policy briefs, could broaden the utility of the framework for diverse stakeholders. Formal evaluation of performance, scalability, and usability in operational public health settings would also strengthen the evidence base for wider adoption.

## Conclusions

This study validates a fully automated Python-based computational pipeline for end-to-end ingestion, analysis, interpretation, and reporting of public health data. By integrating standardised pre-processing, modular analytical components, rule-based narrative interpretation, and programmatic report generation within a single executable workflow, the pipeline demonstrates technical feasibility, internal consistency, and deterministic reproducibility. This framework offers a scalable and reproducible solution for routine public health analytics, training, and operational monitoring, reducing manual effort while improving analytical transparency and standardisation. Future extensions may incorporate additional analytical modules and data sources, further expanding its applicability across diverse public health contexts.

## Figures and Tables

**Figure 5:**
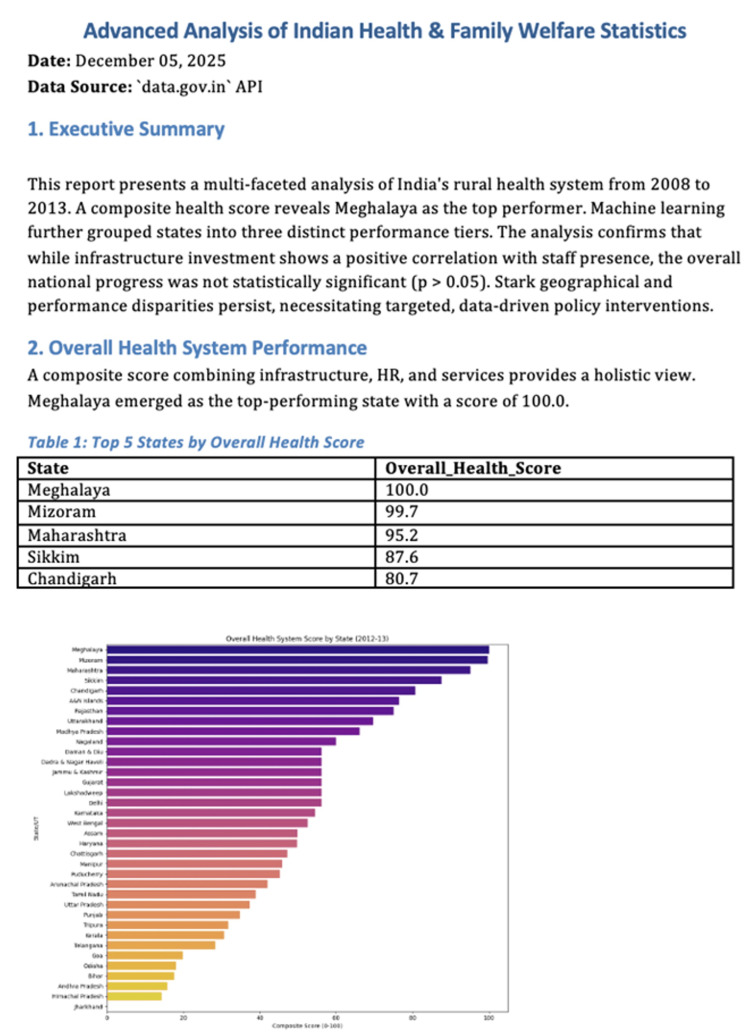
Representative excerpt from the automatically generated Microsoft Word report.
